# CardinalKit: open-source standards-based, interoperable mobile development platform to help translate the promise of digital health

**DOI:** 10.1093/jamiaopen/ooad044

**Published:** 2023-07-19

**Authors:** Oliver Aalami, Mike Hittle, Vishnu Ravi, Ashley Griffin, Paul Schmiedmayer, Varun Shenoy, Santiago Gutierrez, Ross Venook

**Affiliations:** Stanford Byers Center for Biodesign, Stanford University School of Medicine, Palo Alto, California, USA; Department of Epidemiology, Stanford University School of Medicine, Palo Alto, California, USA; Stanford Byers Center for Biodesign, Stanford University School of Medicine, Palo Alto, California, USA; Department of Health Policy, Stanford University School of Medicine; VA Palo Alto Health Care System, Palo Alto, California, USA; Stanford Byers Center for Biodesign, Stanford University School of Medicine, Palo Alto, California, USA; Stanford Byers Center for Biodesign, Stanford University School of Medicine, Palo Alto, California, USA; Stanford Byers Center for Biodesign, Stanford University School of Medicine, Palo Alto, California, USA; Stanford Byers Center for Biodesign, Stanford University School of Medicine, Palo Alto, California, USA

**Keywords:** mobile app development, digital health, open-source software, iOS, android

## Abstract

Smartphone devices capable of monitoring users’ health, physiology, activity, and environment revolutionize care delivery, medical research, and remote patient monitoring. Such devices, laden with clinical-grade sensors and cloud connectivity, allow clinicians, researchers, and patients to monitor health longitudinally, passively, and persistently, shifting the paradigm of care and research from low-resolution, intermittent, and discrete to one of persistent, continuous, and high resolution. The collection, transmission, and storage of sensitive health data using mobile devices presents unique challenges that serve as significant barriers to entry for care providers and researchers alike. Compliance with standards like HIPAA and GDPR requires unique skills and practices. These requirements make off-the-shelf technologies insufficient for use in the digital health space. As a result, budget, timeline, talent, and resource constraints are the largest barriers to new digital technologies. The CardinalKit platform is an open-source project addressing these challenges by focusing on reducing these barriers and accelerating the innovation, adoption, and use of digital health technologies. CardinalKit provides a mobile template application and web dashboard to enable an interoperable foundation for developing digital health applications. We demonstrate the applicability of CardinalKit to a wide variety of digital health applications across 18 innovative digital health prototypes.

## INTRODUCTION

The widespread adoption and proliferation of smartphones and connected devices led to the concept of the “connected” patient. As a result, digital health solutions promise to collect longitudinal health data to engage patients when needed rather than during the traditional episodic clinic visit. Wearable sensors could provide real-time physiologic data to better predict significant clinical changes and passive smartphone data might provide new disease biomarkers for lower cost and scalable diagnosis and monitoring. These are the promises of digital health solutions, and researchers are eager to test their digital health hypotheses and explore their potential. However, significant challenges in building digital health solutions remain, prohibiting groups from transforming a digital health concept into prototypes and products.

There are many complex steps a researcher must complete before being able to launch a minimum viable product (MVP). These include finding a developer, communicating the concept, ensuring stringent HIPAA privacy/security protocols, choosing a mobile application platform and framework, choosing a hosting provider and environment, testing the builds, going through a data risk assessment process as well as an IRB before being able to collect any data. For successful applications, the average development time has been reported to be 15 months, and 47% cost over $200 000 to develop with an average of 15 months to launch (https://research2guidance.com/product/mhealth-economics-how-mhealth-app-publishers-are-monetizing-their-apps/). This extensive process has led to the development of open digital health frameworks that could be used for research or clinical care. For example, Substitutable Medical Apps and Reusable Technologies (SMART) Markers support standards-based data collection of several patient-generated health data instruments through a smartphone app and associated dashboard.^1^ The Learn, Assess, Manage, and Prevent (LAMP) (https://docs.lamp.digital/) platform also provides a framework for integrating digital health data into research or clinical settings for digital psychiatry projects and is being maintained by the Beth Israel Deaconess Medical Center Department of Psychiatry.^2^ Initially designed for neuropsychiatric research, LAMP supports various data types, such as cognitive assessments, screen activity, home time, activity recognition, and sensor integration. A provider-facing dashboard is also available on the LAMP platform, and documentation is readily accessible for developer implementation. UCSF received $9.75 million in NIH funding to develop a digital health platform, called The Eureka Platform (https://www.health-eheartstudy.org), to accelerate digital health.^3^ Approval is required through an intake form, and the UCSF team provides an estimated budget for them to build the project. There are also many enterprise offerings to build digital health solutions. Medable (Palo Alto, CA) provides services to design, build, and host decentralized clinical trials. Zus Medical (Boston, MA) provides a platform of application programming interfaces (APIs) and software development kits (SDKs) to build clinical infrastructure for independent medical groups. This includes an electronic health record (EHR), billing systems, e-prescribing, etc. Commure (San Francisco, CA) provides a platform that unifies data within an entire health care system to provide efficient billing, insights, and interoperability.

Despite the various commercial and academic offerings, there is a continuous need to provide a tool for researchers, case managers, and software developers with lower budgets to more quickly develop compliant mobile applications at a fraction of the cost. In 2018, we open-sourced a comprehensive digital health project named CardinalKit, to support the full ecosystem of emerging digital health data sources and storage providers. The project was inspired by the needs of the simultaneously launched Stanford Biodesign course called Building for Digital Health. In this course, students build projects that were sourced from the School of Medicine clinicians. The functionality provided by CardinalKit enables the prototyping of fully functional digital health applications in one quarter (10 weeks) despite students having little previous mobile application development experience. In this manuscript, we showcase how CardinalKit provides a prebuilt template application addressing several challenges of building digital health applications in a teaching, academic, and industry setting. We provide links to documentation for different components of CardinalKit as a guideline for the reader.

### Mobile application

When CardinalKit was first developed in 2018, a comprehensive ecosystem of mobile iOS development frameworks provided different elements needed in modern digital health applications. ResearchKit provided open-source templated onboarding, consenting, and patient navigation frameworks allowing for both active and passive task assignments. HealthKit provided a centralized health and fitness data repository from the Apple Watch and other connected sensors and devices. CareKit provided the scheduling of tasks, tracking symptoms, and a method to connect with a care team. Apple’s Health application allowed personal EHR data to be stored on the device and made accessible to third-party apps via HealthKit using Health Level Seven International’s (HL7) Fast Healthcare Interoperability Resources (FHIR), an interoperability standard for health data exchange. These comprehensive services made iOS a reasonable choice to start with for the mobile application template.

Nevertheless, these development frameworks come with a learning curve and require significant development expertise and investment in combining and extending their functionality. In addition, a modern mobile application requires several additional web services, including user authentication with third-party sign-in providers, push notifications, and cloud-based data storage, all of which need to be HIPAA-compliant.

**Figure 1.**
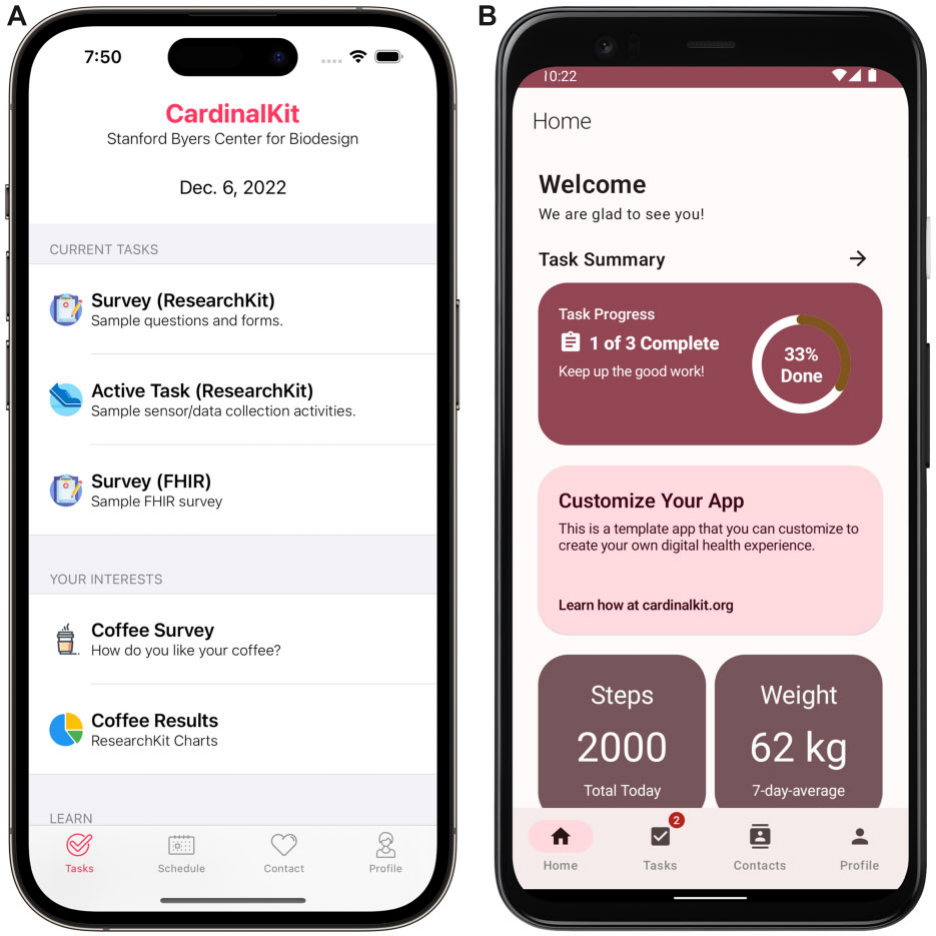
(A) Screenshot of the iOS template application’s main screen. (B) Screenshot of the Android template application’s main screen.

The CardinalKit template application (https://github.com/CardinalKit/CardinalKit) simplifies this process by integrating these components and provides a layer of abstraction, obviating the need for rewriting boilerplate code for each digital health project. This allows development teams to focus their time on building the features that are unique to their project. The template application allows for easy customization of the user flow and content of application components to adapt the template to the specific application domain, as demonstrated in Figure 1A, displaying an example screenshot of a customized task management page. As CardinalKit is an open-source framework, many developers can and have expanded the framework to support additional features and services.

The initial template application has also been expanded to a Kotlin-based Android version of CardinalKit in 2022 (Figure 1B). The Android template application leverages Google’s Health Connect API as a unified and encrypted on-device data store. The Android FHIR SDK enables data storage in the FHIR format and offers FHIR-based questionnaires displayed based on an application designer-defined schedule.

### Cloud service

A HIPAA-ready cloud service is crucial to any modern digital health application, including user authentication, file storage, and a scalable database, all secured with in-flight and at-rest encryption and fine-grained access control. While HealthKit and ResearchKit make it possible to work with health data on iOS devices, they do not include any mechanism for synchronizing these data with a cloud database—a necessity for research and clinical projects. The CardinalKit template application leverages Google Cloud’s Firebase suite of managed cloud services to simplify this process. Firebase is integrated directly into the mobile application using its iOS SDK. CardinalKit serializes data obtained from HealthKit and ResearchKit into the JavaScript Object Notation (JSON), a widely used data interchange format. It then uploads these data to Cloud Firestore, Google Cloud’s scalable NoSQL software as a service (SaaS) document database. CardinalKit represents the data it collects using interoperable schemas, including Open mHealth and HL7 FHIR. This makes it easier to work with data from various sources in the same application and share data with other applications. Google Cloud provides a Healthcare API and FHIR store, a data store that holds FHIR resources, which are compatible with HL7 FHIR RESTful APIs.

While Cloud Firestore is a good option for easily and cost-effectively storing and retrieving various health data types, it does not support the complex queries that are often needed for large-scale data analytics. For this, we use Firebase Extensions to stream data to BigQuery, Google Cloud’s managed data warehouse, which has advanced data analysis and machine learning capabilities. Our web dashboard (described below) also allows survey results to be exported in comma-separated value (CSV) format, which can be imported into most data analysis tools. In future versions of CardinalKit, we aim for the framework to be cloud-agnostic, with the ability to interface with other major cloud providers, including Amazon Web Services (AWS) and Microsoft Azure, as well as self-hosted databases.

## DATA MANAGEMENT AND SYSTEM DESIGN

The CardinalKit platform ties together existing healthcare-ready SDKs, APIs, and frameworks into a comprehensive, yet modular and customizable, package. CardinalKit focuses on providing informed consent, secure data handling, and interoperability. It is up to the application developers who use CardinalKit to provide all content and also to determine how and when the content is delivered. Developers can change and localize all user interface components.

The open-source code, documentation, and example application is provided using the GitHub software version control platform. Developers download and modify the template application in their local integrated development environment (IDE), such as Xcode for iOS and Android Studio for Android development. Developers can distribute the application in any delivery channel they want, including the Apple App Store and TestFlight (Apple Developer Program membership—$99/year) or the Google Play Store (one-time purchase of $25). These platforms can handle automatic updates and staged deployments. Additional costs might arise from the cloud-based deployment of web services or using SaaS platforms.

The CardinalKit application serializes iOS HealthKit and Android Health Connect data points into JSON based on HL7 FHIR and Open mHealth schemas. A multitude of data types can be collected from smartphones and their connected devices. These could include patient-reported outcomes (PROs) in the form of survey responses, heart rate, blood pressure, step counts, geographic location, photos, audio files, and many more data types. The mapping is defined in the CardinalKit FHIR implementation guide (https://cardinalkit.org/cardinalkit-fhir-ig/). This implementation guide is updated as new data types are supported. The connection to external devices to the mobile devices can be achieved using provided Bluetooth connectivity components by the respective operating system-provided APIs. Projects connecting to blood pressure cuffs, smartwatches, as well as chest strap heart monitors based on the CardinalKit template application demonstrate this capability.

CardinalKit is built on transferring “connected patient” data from a smartphone to a cloud storage service, requiring sporadic connectivity to upload data. Connectivity may also be required to receive notifications about scheduled events and for the data to be available to the research coordinator. However, CardinalKit uses offline persistence mechanisms that allow data to be captured, stored locally, and synchronized when a network connection is available. Tasks, schedules, and notifications can also be deployed and persisted locally without the need for an internet connection. These mechanisms reduce the likelihood of data loss due to poor connectivity.

Developers who use CardinalKit are responsible for training their participants on their applications. The template application provides a contact page such that users may contact appropriate resources, whether a study or a clinical program is being deployed. Developers are responsible for customizing this information, performing user testing, and ensuring legal requirements, as each application based on the template contains unique content and configuration of CardinalKit components.

## INTEROPERABILITY

We identify an interoperable system architecture supporting multiple data standards as a core requirement for a reusable and extensible digital health solution. Standardizing digital health data enables integration into EHRs and personal health records to support care teams and patients in disease management and prevention. Additionally, supporting patients in tracking longitudinal digital health data and harmonizing it with disease-specific measures can contribute to improved patient engagement and data-driven shared decision-making. Standards-based approaches for representing and transmitting these various data streams are essential to realizing the value of digital health to improve outcomes.

CardinalKit represents mobile health data according to international healthcare data standards, including Open mHealth^4^ (https://www.openmhealth.org/) and HL7 FHIR (https://www.hl7.org/fhir/). Open mHealth/IEEE 1752 allows health data from wearables and connected medical devices to be modeled using common JSON data schemas that include a clinical context (eg, the distinction between fasting and mealtime blood glucose) and is amenable to high volume, longitudinal measurements due to its simple structure. With the December 31, 2022, Office of the National Coordinator for Health Information Technology (ONC) deadline for the FHIR API implementation by health systems as mandated by the 21st Century Cures Act final rule, mobile applications that are FHIR compatible hold the best chance of being implemented. The CardinalKit framework is “interoperable-ready” from the outset providing the ability to convert all collected data into FHIR resources for storage and data exchange. CardinalKit can also map data between Open mHealth and FHIR, as described in our FHIR implementation guide. CardinalKit is one of the few frameworks which also has extended patient survey data collection and reporting to FHIR with the aid of a web-based survey builder we developed independently of the CardinalKit dashboard. Validated FHIR JSON code for a custom survey can be integrated into CardinalKit iOS and Android template applications from the web-based survey builder. The results are stored as FHIR questionnaire responses and can be exported to an FHIR data store.

### Dashboard

**Figure 2.**
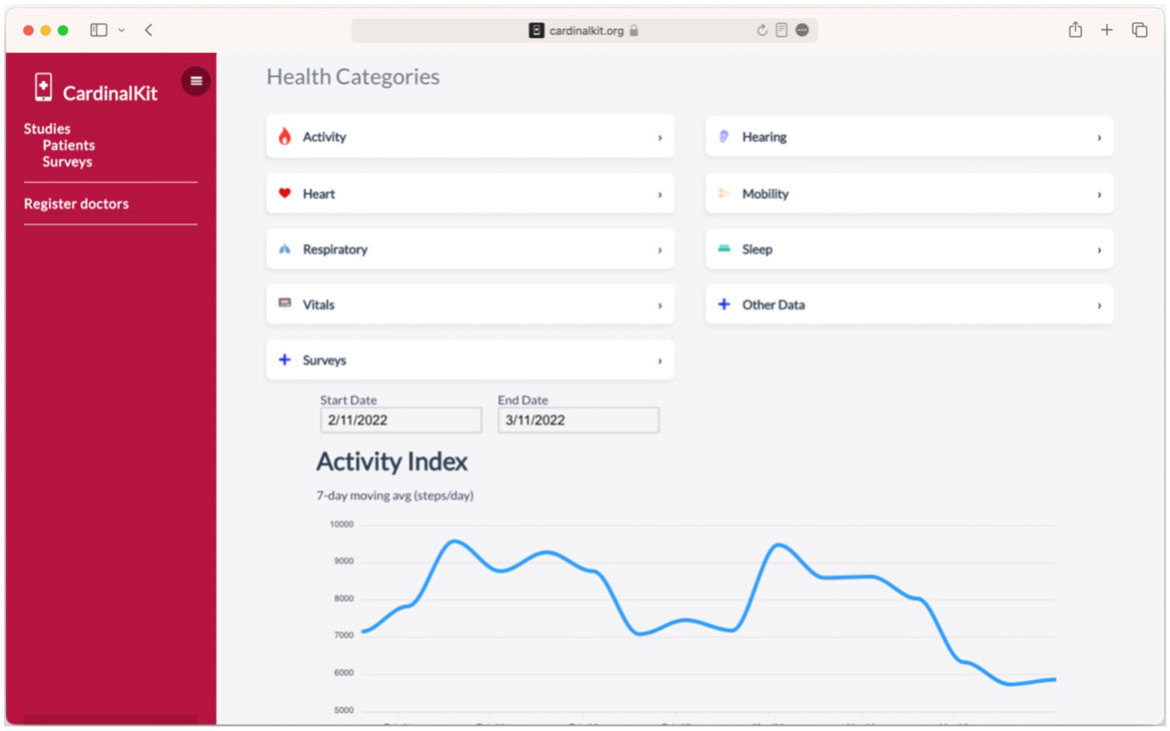
Screenshot of the CardinalKit web dashboard, which allows clinicians and researchers to visualize health data and create and view results from surveys.

Data visualization is essential when running a digital health study, particularly for the study coordinators. These visualizations can indicate if patients are engaging with the study, if they are performing active tasks, and if data are arriving at the web service. Having the ability to review the patient panel data is invaluable and requires focused design and development for each project. CardinalKit provides a simple web dashboard to view survey results as well as graphical visualizations of all HealthKit data elements collected (eg, daily steps, heart rate, etc.; Figure 2).

The web dashboard also supports application developers and researchers. ResearchKit is a well-established framework for building research questionnaires on iOS but requires developers to write surveys directly in code. This makes it challenging for researchers who are not experienced iOS programmers to create and manage surveys. Therefore, the CardinalKit dashboard provides a web-based, no-code survey builder that supports most question types available for the iOS version of the CardinalKit template application. Surveys built with the dashboard are represented in JSON format, saved to the Cloud Firestore database, and downloaded to participants’ devices automatically. This makes it possible to launch new surveys without modifying the application code and pushing an update to the App Store. The web dashboard also contains a scheduler, allowing researchers to choose the start and end dates for a particular survey, including which days of the week each survey will be made available to participants. Survey results can be downloaded from the dashboard in CSV format. They can also be queried directly from the Cloud Firestore database. We plan to expand it to all platforms and upgrade it to an FHIR-based version in the next iteration of CardinalKit.

### Cardinalkit community

Since 2018, over 18 projects have been built using CardinalKit at multiple institutions (Table 1 lists 12 representative projects with descriptions). Most projects were university research projects. Thirty percent have been clinical applications, while 60% were research applications. Several are projects used to build NIH-funded projects, while others have received follow-on funding to support continued development. We have provided design and technical support for each one of these projects to help them come to completion. CardinalKit supports a diverse community, including enterprise products and a high school computer science year-long program in New York.

**Table 1.** A subset of 12 health applications built between 2018 and 2022 using the CardinalKit template application, including the name, description, group of developers, and the current project status

	Application	Description	Developers	Status
1	VascTrac	Cardiovascular passive activity tracking + home 6MTW	Stanford vascular surgeon	Study completed
2	SenseRelief	Apple Watch haptic anti-nausea therapy	Private company/Startup	Launched
3	BUDI	Apple Watch biofeedback upper-limb device for cerebral palsy therapy	Stanford medical and computer science students	Launched
4	Care-IT	End-of-life care documentation (POLST form creation) + patient video end-of-life wish documentation	Stanford Primary Care	Launched
5	Activate	Clinical support tool for individuals with schizophrenia to encourage positive lifestyle changes, including increased activity	Stanford Psychiatry	In development
6	GaitMate	Automated at-home safe, functional mobility assessments used to build predictive models to identify high fall risk characteristics leveraging raw device accelerometer data collection and analysis	Stanford Emergency Medicine	Launched
7	mCHOIR	Integration of sensor data into an open-source learning health system to track and better manage chronic pain syndromes	Stanford Pain Medicine	In development
8	Kidney Care	Outpatient care for post kidney transplant recovery with variable medication tracking	Stanford kidney transplant	In development
9	Slocum Ortho	Using iPhone gait metrics to benchmark functional recovery after ankle fracture.	Slocum Center for Orthopedics and Sports Medicine (Oregon)	Launched
10	LifeSpace	Creates maps of a person’s daily geographic footprint in partnership with the NIH Sponsored REGARDS study using on-device GPS tracking	Stanford Epidemiology	Launched
11	MyOIT	Oral immunotherapy study	Sean N. Parker Center for Allergy and Asthma Research	In development
12	High School Course Projects	Year-long projects for students to learn to code and conduct scientific research	Walt Whitman High School (New York)	Launched

The CardinalKit.org website is the portal to our community where one can join our mailing list or Slack team, view sample projects, access documentation, and request technical support. Our team hosts workshops throughout the year where developers can learn how to get started with CardinalKit and ask questions about their projects. Many inbound requests for CardinalKit interest arise from the complexities of existing frameworks (eg, ResearchKit).

Our Slack channel is the hub of communication for the CardinalKit community (https://cardinalkit.org), with over 200 members from the digital health ecosystem. Our community uses Slack to coordinate features and code development, discuss commercial and academic opportunities and happenings, and for members to connect and collaborate.

## DISCUSSION

Open-source digital health platforms such as CardinalKit provide a foundation of prebuilt solutions for developing mobile digital health projects. A growing body of evidence demonstrates the value of digital health tools to detect physiological changes and improve the management of numerous chronic conditions.^5–10^ Notably, patients are gaining more access to their health data due to federal US regulations (eg, 21st Century Cures Act, Interoperability and Patient Access final rule) and the proliferation of mobile devices. However, very few tools enable patients to curate their data from various sources, derive insights, and share data with their care team or researchers. Developing such tools requires careful consideration of data privacy, storage, standards, usability, and analytic pipelines for knowledge discovery.

Extending and integrating existing solutions into larger-scale clinical or research systems usually requires extensive remodeling and changes to existing infrastructure. While this approach provides a starting point, feedback from CardinalKit developers and experience building several projects using digital health frameworks showcased drawbacks when extending the tightly integrated application templates. We argue that an approach building on interchangeable, modular components enables greater extensibility and enables systems to use partial functionality required for their specific use case. Applying this modularization is the next step for reusable digital health solutions encouraging community contributions by selectively adding, maintaining, and sharing functionality interacting with well-defined extension points within mobile applications, dashboards, and cloud services. Therefore, we plan to extend the functionality, extensibility, and reusability of CardinalKit by further modularizing the framework into smaller reusable components. A clear separation of concerns within the framework will enable application developers to reuse CardinalKit in more application and research domains. Health data standards such as HL7 FHIR will build the foundational data format to exchange information between these components. We plan to introduce four main extension points to enable data source modules, data storage providers, user interface modules, and research application interface modules. We aim for eventual feature parity between the iOS and Android instantiations of CardinalKit, including FHIR questionnaires, mobile data collection using Google Health Connect, and an extensible upload to different data storage providers.

Additionally, we plan to develop a SMART on FHIR implementation of the dashboard to allow integration with EHRs.^11^ Historically, external clinical decision support (CDS) tools have not been as widely used as today, as many have been standalone applications outside the EHR.^12^ This requires additional login authentication, duplicate data entry already captured in the EHR and can disrupt clinical workflows. Integrating digital health data within existing clinical processes provides easier access to rich contextual lifestyle data, which could be used to help explain or predict health-related outcomes. The dashboard will likely require enhancements that support clinical decision-making from these multimodal and time-varied data streams (eg, visual analytics tools, clinically meaningful references, CDS hooks). An essential aspect of the development of CardinalKit and the future extension points is a vibrant open-source community. Building CardinalKit in the open and encouraging contributions promotes our development philosophy for digital health applications and resulted in several useful community-based contributions. Open-source digital health platforms enable the creation of tools at a fraction of the traditional software development cost. These projects can only flourish if used and maintained. For example, CardinalKit’s regular use in Stanford Biodesign’s Building for Digital Health courses helps maintain and update the codebase. The modularization of different CardinalKit components will further encourage developers to build, maintain, and steer useful extension points that numerous CardinalKit-based applications can integrate and benefit from. We believe this approach can embrace several existing digital health solutions, such as LAMP and CardinalKit, integrating them into a larger modularized digital health ecosystem.

## CONCLUSION

Realizing the potential for personalized and precision health through digital health applications remains a desirable goal to increase access to health care, lower costs, and improve the experience by meeting patients where they are. Significant cost and effort are required to build, deploy, and study applications. An open-source, standards-based, interoperable mobile development framework such as CardinalKit can help accelerate this cause. We demonstrate the applicability of CardinalKit to a wide variety of digital health applications across 18 innovative digital health prototypes. We aim to further modularize and extend the functionality of the framework, template application, and dashboard in the future.

## Data Availability

We have included links to the open-source CardinalKit framework in this manuscript. Further information and access to all documentation and source data can be accessed at: https://cardinalkit.org/
